# Transcriptomic Analysis Reveals the Sexually Divergent Host–*Wolbachia* Interaction Patterns in a Fig Wasp

**DOI:** 10.3390/microorganisms9020288

**Published:** 2021-01-31

**Authors:** Hong-Xia Hou, Dan Zhao, Jin-Hua Xiao, Da-Wei Huang

**Affiliations:** Institute of Entomology, College of Life Sciences, Nankai University, Tianjin 300071, China; houhongxia003@126.com (H.-X.H.); zd15110606011@126.com (D.Z.)

**Keywords:** interaction, *Wolbachia*, sexually differential expression, fig wasp

## Abstract

*Wolbachia* are widely distributed in arthropods and nematodes, acquiring nutrients from the hosts, and inducing remarkable reproductive modulations on the hosts. To investigate the interaction of *Wolbachia* and insects, *Wolbachia* are often artificially eliminated from *Wolbachia*-infected hosts, which may produce negative effects of antibiotics. In the present study, based on the transcriptomic data of a fig wasp species *Ceratosolen solmsi* with two sibling lineages, one natively infected and the other noninfected with *Wolbachia*, we investigated the expression patterns of genes. The comparison results of differently expressed genes (DEGs) between *Wolbachia* infected and noninfected samples show that males have many more DEGs than females. The male unique upregulated genes are enriched in biological processes mainly related to biosynthesis, transport, positive regulation of I-kappaB kinase/NF-kappaB signaling, MAPK cascade, and pathogenesis; the male unique downregulated genes are enriched in biological processes mainly related to transport, oxidation–reduction, cellular responses to oxidative stress, lipid oxidation, cytoskeleton organization, actin filament-based process, and localization. In addition, for the *Wolbachia*’s gene expression, the number of genes up-regulated in males is higher than that in females. The results revealed divergent patterns of the host–*Wolbachia* interactions between males and females in the fig wasp species.

## 1. Introduction

The maternally inherited alpha-proteobacteria *Wolbachia pipientis* is an obligate endosymbiotic bacterium and has a widespread and global distribution in filarial nematodes and diverse arthropod hosts [1]. To increase the transmission and propagation, *Wolbachia* induces a wide range of physiological manipulations in the hosts, including the modulation of the reproductive system, such as the cytoplasmic incompatibility, feminization, parthenogenesis, and male killing [1]. In recent years, various studies have shown that *Wolbachia* can be beneficial to the insect hosts in diverse aspects, such as increasing the hosts’ fecundity, longevity and survival, nutritional provision, and enhancing the hosts’ antiviral ability [2]. In some cases, *Wolbachia* show influences on the genomic divergence of hosts’ mitochondria, implicating the speciation of hosts [3]. Some recent studies have also tried to understand the interactions between *Wolbachia* and insect hosts in the aspect of gene expression. For example, in *Drosophila*, host genes, age, tissue, genotype, and virus infection can regulate the titer of *Wolbachia* [4,5,6,7]. Additionally, the regulation of one or two genes in the *Wolbachia* can cause remarkable phenotypic influence such as cytoplasmic incompatibility (CI) on the hosts [8,9]. To better understand the biological interaction between a mosquito, *Aedes fluviatilis*, and its native infected *Wolbachia*, a study was designed to compare the transcriptomes of the mosquitos infected and noninfected with *Wolbachia*, in which the samples treated with tetracycline were set as the *Wolbachia*-non-infected control group [10]. There have been various studies focusing on the interaction between host and *Wolbachia*, however, few studies have underscored the putative divergent patterns of host–*Wolbachia* interaction in each gender of the hosts.

To investigate the interactions between *Wolbachia* and insect hosts, *Wolbachia* is usually artificially eliminated from the *Wolbachia*-infected hosts through antibiotic treatment. However, antibiotic treatment may produce negative effects to the hosts which interfere with the final analyses, such as changing the composition of the gut microbes of the hosts, producing a strain-specific response to antibiotics and *Wolbachia* rebounds appearing [11,12]. If an insect model with a native distinction of *Wolbachia* infection (infected or noninfected) between different individuals within the same species, or between two closely related sibling species can be used, we can accurately infer the influences of *Wolbachia* on the hosts.

Inside the compact inflorescences (the syconia) of fig trees (*Ficus*: Moraceae), there lives a group of hymenopteran insects, the fig wasps, in which the “fig pollinators” (Agaonidae: Chalcidoidea) have had an obligate pollinating mutualism with fig trees for ca. 65 million years [13,14]. Previous studies established that the fig wasps are infected with *Wolbachia* in the whole range of the particular species [15]. On the fig tree of *Ficus hispida* in China, we detected two sibling lineages of fig pollinator species *Ceratosolen solmsi*, presenting distinct association with *Wolbachia* infection, with one group natively infected but the other noninfected [16]. They are almost indistinguishable by their morphological characteristics. This fascinating native divergence of *Wolbachia* infection between the two sibling lineages of *C. solmsi* makes them an ideal model to proximately infer the reciprocal influences of *Wolbachia* on each gender of the hosts.

In this study, transcriptomic sequencing of the fig wasp species *C. solmsi* was used to detect the sexually different expression patterns of genes that may be associated with the *Wolbachia*–host interaction patterns. We obtained the global gene expression data of fig wasps of each gender which contained *Wolbachia* infected and noninfected samples, as well as the differently expressed genes of *Wolbachia* between both genders of *Wolbachia* infected fig wasp lineage. Our results show that whether in the fig wasps or *Wolbachia*, there are sexually different expression patterns, which imply different interaction patterns of the host–*Wolbachia* between both genders of the fig wasp *C. solmsi.*

## 2. Materials and Methods

### 2.1. Sample Preparation

We detected natural females of *Wolbachia*-infected (*Cera*^+^) and *Wolbachia*-noninfected (*Cera*^−^) fig wasp lineages of *Ceratosolen solmsi*, and then respectively inoculated them on their host fig trees of *Ficus hispida* (*Ficus*: Moraceae). The inoculation experiment was completed as previously reported [17] in Danzhou, Hainan province, China from June to August in 2014. We collected all the female “daughter” and male “son” wasps for further studies. For the RNA sequencing analyses, altogether four sample groups were set: *Cera*^+^ female, *Cera*^+^ male, *Cera*^−^ female, and *Cera*^−^ male. Three biological replicates were also set for each sample group. In each replicate experiment, 50 wasps were used for RNA extraction.

### 2.2. Libraries Construction and Sequencing for RNA Samples

Total RNA was isolated using the EasyPure RNA kit (*TransGen*, Beijing, China) and treated with DNase I (*TransGen*, Beijing, China). A NanoDrop ND-1000 Spectrophotometer (*Nano-Drop Technologies*, Wilmington, DE, USA) was used to confirm adequate RNA concentration and A260/A280 ratio. At least 3 μg RNA per sample was used for further studies. The host mRNA was enriched by poly-T oligo-attached magnetic beads. To enrich the mRNA of the endosymbiotic bacterium *Wolbachia*, both prokaryotic and eukaryotic rRNA were first deleted from the total RNA, and then host mRNA were excluded from the rest RNA using poly-T oligo-attached magnetic beads. The NEBNext^®^ UltraTM RNA Library Prep Kit for Illumina^®^ (NEB, Ipswich, MA, USA) was used to construct the library, and the Agilent Bioanalyzer 2100 system was used to assess the quality of the library. The eligible library was sequenced on the Illumina Hiseq platform, and produced 125 bp paired-end reads finally.

### 2.3. Gene Expression Patterns between Both Genders of Cera^+^ and Cera^−^ Lineages

We sequenced altogether twelve samples. To quantify gene expression level, we mapped RNA-seq clean reads to the genome (unpublished data in our lab) using Tophat2 (http://ccb.jhu.edu/software/tophat/index.shtml) and normalized the value using FPKM (Fragments Per Kilobase of transcript sequence per Millions base pairs sequenced) for the samples. Then differentially expressed genes were calculated using DESeq (http://www.bioconductor.org/packages/release/bioc/html/DESeq.html) for the four groups, *Cera*^−^ female between *Cera*^−^ male, *Cera*^+^ female between *Cera*^+^ male, *Cera*^−^ female between *Cera*^+^ female, and *Cera*^−^ male between *Cera*^+^ male. In order to explore if there were any functional enrichments for DEGs, GO enrichment analyses were implemented using topGO (http://www.bioconductor.org/packages/release/bioc/html/topGO.html).

### 2.4. Real Time qPCR Validation of the Differentially Expressed Wolbachia Genes between Both Genders of Cera^+^ Lineage

Total RNAs were extracted for the sample of each gender of *Cera*^+^ lineage (25 specimens for each sample) by using *TransZol* Up Plus RNA Kit (*TransGen*, Beijing, China), with each sample containing three biological replicates. For each sample, cDNAs were reverse-transcribed from 300 ng total RNAs by using TransScript^®^ One-Step gDNA Removal and cDNA Synthesis SuperMix (*TransGen*, Beijing, China). Real time qPCR experiments were demonstrated by using TransStart^®^ Top Green qPCR SuperMix (+Dye II) (*TransGen*, Beijing, China) on the system of Agilent Mx3000P (USA), with *groEL* gene used as the reference gene. The primers are listed in Supplementary Table S1. The expression difference and significance of each gene was calculated based on 2^−ΔΔCT^.

## 3. Results

### 3.1. Differential Expression Analysis of the Fig Wasps in Two Directions: “Wolbachia-Infection Associated DEGs” and “Gender Associated DEGs”

To get a global understanding on the gene expression patterns between *Wolbachia*-infected lineage (*Cera*^+^) and *Wolbachia*-noninfected lineage (*Cera*^−^), we set four sample groups to make comparisons based on the RNA sequencing data: *Cera*^+^ female versus *Cera*^−^ female, *Cera*^+^ male versus *Cera*^−^ male, *Cera*^−^ female versus *Cera*^−^ male, and *Cera*^+^ female versus *Cera*^+^ male. We compared the differentially expressed genes (DEGs) in more detail in two directions: “*Wolbachia*-infection associated DEGs” (in calculation of the gene expression in each gender, the *Cera*^−^ sample is set as the control and the *Cera*^+^ sample as the treatment), and “Gender associated DEGs” (in calculation of the gene expression within each lineage, the male sample is set as the control and the female sample as the treatment) (Table 1).

As shown in Table 1, for “*Wolbachia*-infection associated DEGs”, in the comparison between females of *Cera*^+^ and *Cera*^−^, we detected only a small quantity of differentiation (only 556 DEGs, with differentiation gene proportion of 3.8%), while in the comparison between males of *Cera*^+^ and *Cera*^−^, the number of differentiated genes was 2797 (differentiation gene proportion of 19.2%), which indicates that the infection of *Wolbachia* may result in having a much more significant effect on the expression of genes in males than in females. For “Gender associated DEGs”, compared to the large gene expression differentiation between both genders in the *Cera*^−^ lineage (4486 DEGs, with differentiation gene proportion of 30.8%), the sexually differently expressed genes decreased in the *Cera*^+^ lineage (2555 DEGs, with differentiation gene proportion of 17.5%), indicating a *Wolbachia* associated decrease in the sexual differentiation of gene expression.

### 3.2. Differential Expression Patterns of the “Wolbachia-Infection Associated DEGs” between Both Genders of Fig Wasps

**Figure 1:**
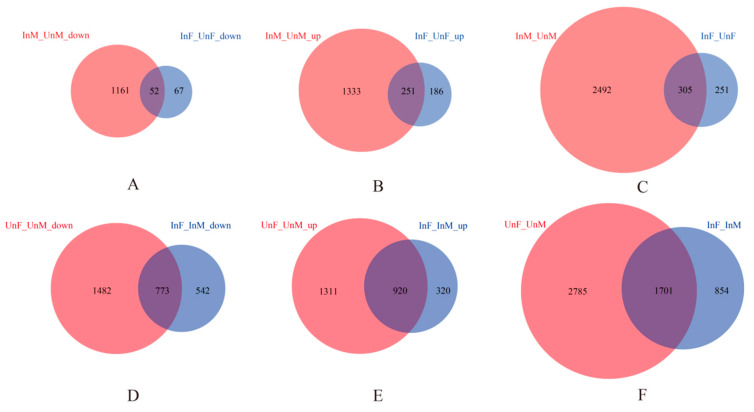
The differentially expressed genes (DEGs) analyses. We compared the “*Wolbachia*-infection associated DEGs” and the “Gender associated DEGs”. (1) In the comparison of “*Wolbachia*-infection associated DEGs” (**A**–**C**) between *Cera*^+^ and *Cera*^−^ samples in each gender, by setting the *Cera^−^* samples as the controls, we classified the DEGs into three groups: the downregulated, upregulated, or unclassified genes in *Cera*^+^ samples. There are thus three groups of DEGs in males: downregulated in *Cera*^+^ male (InM_UnM_down), upregulated in *Cera*^+^ male (InM_UnM_up), and DEGs between *Cera*^+^ and *Cera*^−^ males (InM_UnM), and the same three groups of DEGs in females: downregulated in *Cera*^+^ female (InF_UnF_down), upregulated in *Cera*^+^ female (InF_UnF_up), and DEGs between *Cera*^+^ and *Cera*^−^ females (InF_UnF). For each of the three groups of DEGs, we then compared the overlapping pattern between both genders, and obtained shared DEGs in both genders and the gender-specific DEGs. For example, as Figure 1A shows, in the downregulated DEGs in *Cera*^+^ samples, there are 1161 genes unique downregulated in males, 67 unique in females, and 52 shared in both genders. (2) In the comparison of “Gender associated DEGs” (**D**–**F**), between both genders’ samples in *Cera*^+^ or *Cera*^−^ lineage, there are also three groups of DEGs in each lineage: downregulated in female, upregulated in female, and DEGs between both genders. For each of the three groups of DEGs, the overlapping pattern between *Cera*^+^ and *Cera*^−^ lineages were compared, and the shared DEGs, lineage-specific DEGs were obtained.

**Figure 2:**
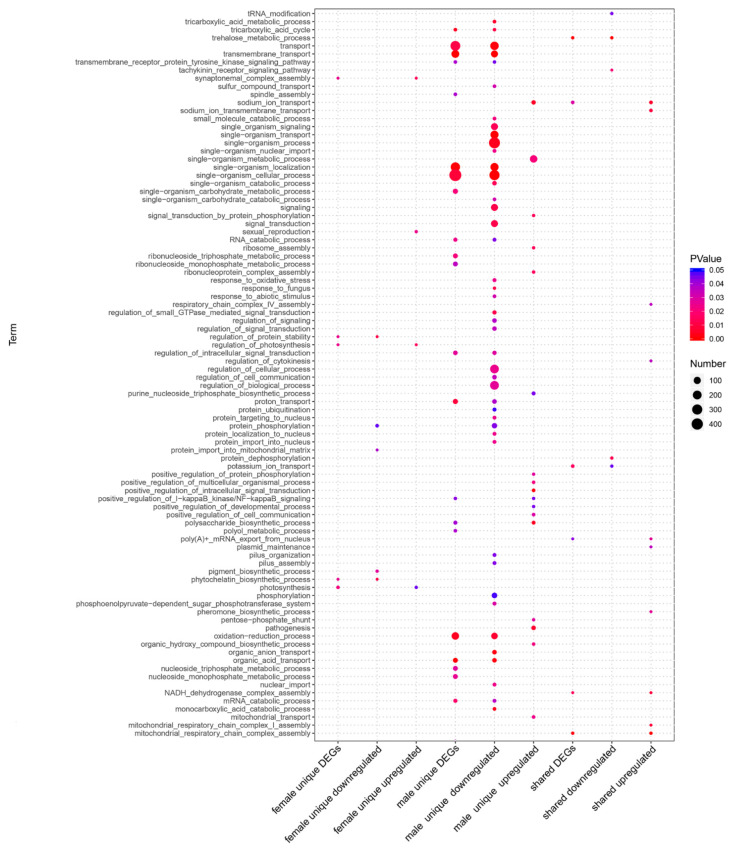

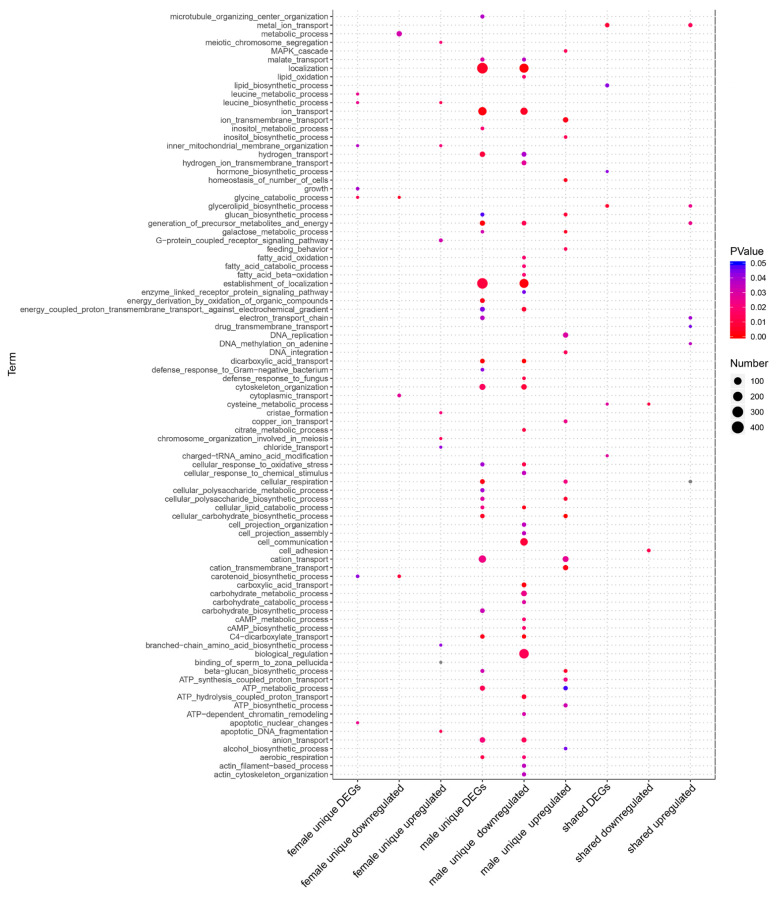
Biological processes of GO enrichment analysis for the “*Wolbachia*-infection associated DEGs”. The results show that males have the most significantly enriched GO items for the DEGs (*p* value < 0.05).

In the comparison of “*Wolbachia*-infection associated DEGs”, we had nine groups of DEGs (control: *Cera*^−^, treatment: *Cera*^+^): male unique downregulated genes, male unique upregulated genes, male unique DEGs, female unique downregulated genes, female unique upregulated genes, female unique DEGs, shared (by both genders) downregulated genes, shared upregulated genes, and shared DEGs. Compared to females, males have the most DEGs (Figure 1A–C). We demonstrated GO enrichment analyses on all the nine groups of DEGs (with the enriched biological processes shown in Table S2), and the results of the significantly enriched biological processes (*p* value < 0.05) items show that males have the majority of enriched GO items for the DEGs (Figure 2). The male unique upregulated biological processes were enriched in 37 GO terms, mainly including biosynthesis (biosynthesis of carbohydrate, polysaccharide, beta-glucan, inositol, ATP, purine nucleoside triphosphate and alcohol, DNA replication and integration, ribosome assembly and ribonucleoprotein complex assembly etc.), transport (ions and mitochondria), positive regulation of I-kappaB kinase/NF-kappaB signaling, MAPK cascade, and pathogenesis. The male unique downregulated biological processes were enriched in 80 GO terms, mainly including transport (carboxylic acid, ions, sulfur compound, and energy coupled proton transmembrane transport, and protein import into nucleus), oxidation–reduction, cellular responses to oxidative stress, lipid oxidation, cytoskeleton organization, actin filament-based process, localization, tricarboxylic acid cycle (TAC), ATP-dependent chromatin remodeling, and protein ubiquitination. The females had much less enriched GO items for the DEGs. The female unique upregulated biological processes were enriched in 14 GO terms, mainly including apoptotic DNA fragmentation, leucine biosynthetic process, meiotic chromosome segregation, inner mitochondrial membrane organization, G–protein coupled receptor signaling pathway, and chloride transport; the female unique downregulated biological processes were enriched in nine GO terms, mainly including biosynthesis (carotenoid, phytochelatin, pigment), glycine catabolic process, regulation of protein stability, protein phosphorylation, and cytoplasmic transport.

### 3.3. Differential Expression Patterns of the “Gender Associated DEGs” between Both Lineages of Fig Wasps

In the comparison of “Gender associated DEGs” (Figure 1D–F), we also had nine groups of DEGs (control: male, treatment: female): *Cera*^−^ unique downregulated genes, *Cera*^−^ unique upregulated genes, *Cera*^−^ unique DEGs, *Cera*^+^ unique downregulated genes, *Cera*^+^ unique upregulated genes, *Cera*^+^ unique DEGs, shared downregulated genes (by both sibling lineages), shared upregulated genes, and shared DEGs. Overall, the *Cera*^−^ lineage had more “Gender associated DEGs” than the *Cera*^+^ lineage. For all these “Gender associated DEGs”, we also demonstrated GO enrichment analyses (Table S3), and due to the large number of sexual DEGs, we obtained a respectable large number of GO enrichment items, in which the processes of metabolism and transport were the most distinct differential between both genders, similar to what we discovered in previous studies [14]. The decrease of differences between both genders in *Wolbachia*-infected lineage may be associated to *Wolbachia*, which deserve further investigation.

### 3.4. The Sexually Different Expressed Genes of Wolbachia in Cera^+^ Lineage

From the above studies on the gene expression of fig wasp hosts, we obtain the information that each gender of the host has different gene expression patterns that may be associated to the infection of *Wolbachia*. Considering the intimate relationship between *Wolbachia* and host, we expect distinct gene expression patterns of *Wolbachia* in each gender of *Cera*^+^ lineage. However, the endosymbiotic feature of *Wolbachia* impedes its isolation from the host, and thus the investigation of its gene expression pattern by high-throughput methods. Despite the fact that we tried to get the preliminary information on the expression pattern of *Wolbachia* genes by sorting out the *Wolbachia*’s sequencing reads from the high-throughput sequencing results. Since the hosts predominate the mRNAs, we could not get a saturated data for the *Wolbachia* mRNAs even if we sequenced 25 Gb data for each sample. Even though, based on these data, we could not calculate the correct expression reads of each *Wolbachia* gene, we could still compare its divergent expression patterns among different samples, which we could further validate by qPCR experiments.

**Figure 3:**
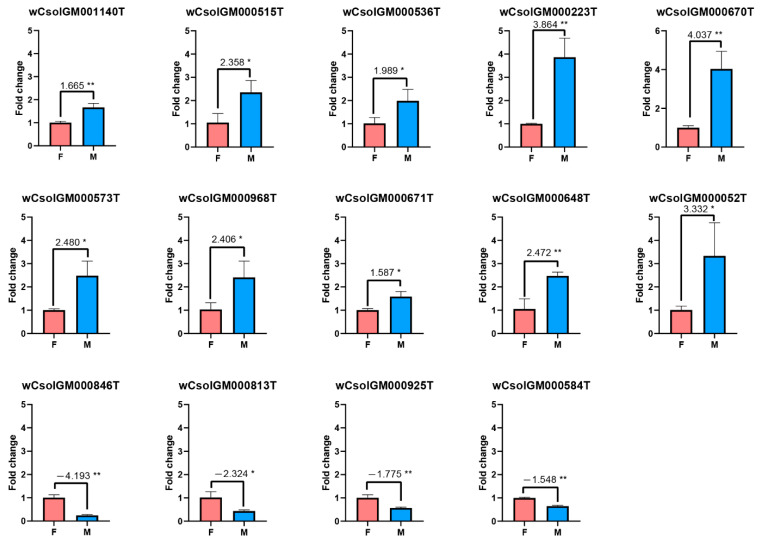
RT-qPCR assay of differentially expressed *Wolbachia* genes in female and male hosts. F: Female (control); M: Male (treatment); *: 0.01 < *p* value < 0.05; **: *p* value < 0.01.

The results show that 53 *Wolbachia* genes are significantly sexually-differentially expressed in *Cera*^+^ lineage, in which we choose some highly divergent genes (20 up-regulated genes and six down-regulated genes in males) for further experimental validations. By experimental validation, we finally obtained ten significantly up-regulated and four significantly down-regulated genes in males (Figure 3, Table 2, the other experimentally validated genes, with the expression differentiation patterns almost similar to the results of high-throughput data but not significant, are not listed). The ten up-regulated genes are listed as below: genes that may encode a putative DNA recombinase, a transposase, and a transferase, a gene of the bacterial phage WO that may function in DNA passage, a hsp70 gene, a hsp40 gene and its co-chaperone hscB, a gene in the toxic component of the toxin–antitoxin module, a gene related to isoprenoid biosynthetic process, and a cell cycle-aspartyl protease family gene. The four down-regulated genes include a cell cycle control gene (*CtrA*), an actin-binding gene, a ribosome biogenesis gene and a transcription elongation factor gene (*GreA*).

## 4. Discussion

In this study, based on the two sibling lineages of the fig wasp species *Ceratosolen solmsi*, one natively infected with *Wolbachia* and the other noninfected, we explored whether the different gene expression patterns between both genders of the fig wasps are related to the *Wolbachia*–host interaction patterns, and compared the expressions of *Wolbachia* genes in both genders of hosts infected with *Wolbachia*.

### 4.1. The Expression Patterns of the “Wolbachia-Infection Associated DEGs” Show Great Differences between Males and Females

Our comparative study focused on comparing the differences in gene expression between samples of *Cera*^+^ and *Cera*^−^, based on females and males of the fig wasp hosts. We detected that male wasps display more significant DEGs than females. GO enrichment on the male-specific DEGs showed that the males of *Cera*^+^ may increase the synthesis and transport of many metabolites, to restrict the presence of *Wolbachia* [7], or for the utilization of themselves or the parasite *Wolbachia*. Furthermore, in the males of *Cera*^+^, the oxidation–reduction process is downregulated, whereas the MAPK cascade and the positive regulation of I-kappaB kinase/NF-kappaB signaling are upregulated. These results indicate that the males cannot control the ROS homeostasis disturbed by *Wolbachia,* thus the accumulation of ROS increases the expression of MAPK signaling and the transcription factor nuclear factor-kB [18]. The downregulated cytoskeleton organization and actin filament-based process may be a signal that the male hosts are trying to resist the transport of *Wolbachia*, or *Wolbachia* has controlled the cytoskeleton organization for their own use. The upregulated transcription factor nuclear factor-kappaB and pathogenesis imply that the male hosts may be trying to resist or accommodate the infection of *Wolbachia* by increasing the innate immunity level [19], or the *Wolbachia* may improve the ability of hosts to resist pathogens [20].

It is noteworthy that both genders have some shared upregulated and shared downregulated genes between the samples of *Cera*^+^ and *Cera*^−^. Interestingly, the shared upregulated genes are mainly enriched in the function of mitochondrial complex I, III, and IV, all of which are respiratory chain complexes with the proteins encoded by both nuclear and mitochondrial genes. For example, the protein encoded by gene177 is a NADH dehydrogenase ubiquinone iron–sulfur protein 4 (NDUFS4) that may extensively interact with almost all components of the matrix arms in Complex I of mitochondrion [21]. In contrast, for the shared downregulated genes in both genders, besides some membrane-associated genes, we also noticed two genes in the trehalose metabolic process. In insects, trehalose are important not only for energy supply, but also for the stabilization of cellular membrane and chitin synthesis [22]. In fig wasp species, the GO term of the trehalose metabolic process includes three genes: two forms of trehalase (Gene IDs in *Cera*^+^ lineage: evm.model.scaffold0.1523 and evm.model.scaffold5.179) involved in the hydrolysis of trehalose, and the trehalose-6-phosphate synthase (Gene ID in *Cera*^+^ lineage: evm.model.scaffold38.125) involved in trehalose synthesis. Compared to *Cera*^−^ lineage, the downregulation of evm.model.scaffold0.1523 and evm.model.scaffold38.125 in both genders of the *Cera*^+^ lineage most promisingly suggests that by decreasing the metabolism of trehalose, *Wolbachia* may have inhibited the ability of the fig wasp host in the regulation of chitin biosynthesis or membrane stabilization, for their own easier transmission across the hosts’ cells. Interestingly, in the pheromone metabolism process, we detected a shared upregulated gene involving the biosynthetic process (Gene ID in *Cera*^+^ lineage: Novel02068), while a shared downregulated gene in pheromone sensitivity (Gene ID in *Cera*^+^ lineage: evm.model.scaffold8.156, encoding sensory neuron membrane protein 1) [23], may hint to a damp on pheromone response in *Cera*^+^ lineage.

In summary, in comparison of the gene expression between the samples of *Cera*^+^ and *Cera*^−^, the male wasps display much more significant DEGs and more significantly enriched biological processes than females. The male hosts may restrict or adapt to the presence of *Wolbachia* by synthesizing and transporting many metabolites, and upregulating the MAPK cascade and the I-kappaB kinase/NF-kappaB signaling to control the ROS homeostasis. Meanwhile, the male hosts may down-regulate the cytoskeleton organization to resist the transport of *Wolbachia*.

### 4.2. The Sexually Differently Expressed Genes of Wolbachia are Related to Different Interaction Patterns with Their Hosts

When we compared the expression of *Wolbachia* genes in both genders of the *Cera*^+^ lineage, we detected a pattern that gender-biased expression of *Wolbachia* genes are mostly upregulated in males, which is similar to the results in *Drosophila melanogaster* [24]. In the female hosts, we found a *GreA* gene (wCsolGM000925T) and an actin binding gene (wCsolGM000813T) are upregulated, which indicate that the *Wolbachia* have a better ability to resist oxidant stress and to transfer along the hosts’ cytoskeleton in the female hosts (Figure 3, Table 2) [25]. While in the male hosts, we found ten genes (wCsolGM001140T, wCsolGM000515T, wCsolGM000536T, wCsolGM000223T, wCsolGM000671T, wCsolGM000670T, wCsolGM000573T, wCsolGM000052T, wCsolGM000648T, wCsolGM000968T) are upregulated, which are mainly associated with DNA duplication, oxidative stress, virulence modulation, and cell cycle (Figure 3, Table 2). With the addition of the downregulation of the *CtrA* gene (wCsolGM00846T) in the male hosts, we speculate that *Wolbachia* in the male hosts may undergo the following processes: (1) increased DNA replication, revealed by the upregulation of DNA recombinase and DNA repair; (2) more active modulations to the oxidative stress and virulence of the hosts; (3) regulation of the cell cycle, especially the blocking of cell division. The different gene expression profiles of *Wolbachia* genes between the both genders suggest different interaction patterns of *Wolbachia* with their hosts. In the male hosts, which may be the dead end of *Wolbachia*, *Wolbachia* have more up-regulated genes, and they may act on the hosts via virulence modulation. Meanwhile, they may preserve their cells integrity through launching a self-protection process with more DNA replication but with delayed cell divisions to compensate against the host attenuation [25]. All of these suggestions deserve further investigation, which may help uncover the interaction between *Wolbachia* and their insect hosts.

## 5. Conclusions

In this study, to understand the sexually different expression patterns of genes associated with the *Wolbachia*–host interaction patterns, we performed comprehensive comparable studies on the global gene expression patterns of the fig wasp hosts and the endosymbiotic bacteria *Wolbachia* based on the transcriptomic data. The results show great differences of gene expression patterns between females and males that may be associated with the presence of *Wolbachia*. Compared to the females, the males presented many more differentially expressed genes (DEGs) between *Wolbachia*-infected and *Wolbachia*-noninfected samples, and the function of many DEGs may be associated with the infection of the endosymbiotic bacteria *Wolbachia*. In the *Wolbachia*-infected samples, the expression of *Wolbachia* genes also showed sexually different expression patterns. There are more up-regulated *Wolbachia* genes in the male hosts than female hosts, which are also closely related to the *Wolbachia*–host interaction. These results suggest different interaction patterns of host–*Wolbachia* between both genders of fig wasps, which may help in further investigation of the molecular mechanism of the interaction between *Wolbachia* and their hosts.

## Figures and Tables

**Table 1:** Comparisons of the Differentially Expressed Genes (DEGs) of the fig wasps. *Cera*^−^: the fig wasp lineage noninfected with *Wolbachia*, *Cera*^+^: the fig wasp lineage infected with *Wolbachia*, F: female, M: male (e.g., *Cera*^−^F indicates female wasps with no infection of *Wolbachia*); Upregulated gene number: the gene number upregulated in treatment when compared to the control; Downregulated gene number: the gene number downregulated in treatment when compared to the control; Total DEGs number: the summary of the up- and down-regulated genes; Total gene number: the total gene number analyzed in the study; Proportion of DEGs: the division of the total DEGs number to the total gene number.

Compared Group (Treatment: Control)		Upregulated Gene Number	Downregulated Gene Number	Total DEGs Number	Total Gene Number	Proportion of DEGs
*Wolbachia*-infection associated DEGs	*Cera*^+^F: *Cera*^−^F	437	119	556	14562	3.8%
	*Cera*^+^M: *Cera*^−^M	1584	1213	2797	14562	19.2%
Gender associated DEGs	*Cera*^−^F: *Cera*^−^M	2231	2255	4486	14562	30.8%
	*Cera*^+^F: *Cera*^+^M	1240	1315	2555	14562	17.5%

**Table 2:** Information on the validated *Wolbachia* genes that are differentially expressed between female and male hosts samples. (Male: treatment; Female: control).

Gene ID	Functional Annotation	Response of *Wolbachia* to the Host	Different Expression
wCsolGM001140T	Site-specific DNA recombinase.	Increased DNA duplication in males.	Up-regulated
wCsolGM000515T	An ortholog of wHa_08300, putative transposase.		
wCsolGM000536T	A gene of the bacterialphage WO. The encoded protein forms a hole, or portal, that enables DNA passage during packaging and ejection; it also forms the junction between the phage head (capsid) and the tail proteins; it functions as a dodecamer of a single polypeptide of average mol. wt. of 40–90 kDa.		
wCsolGM000223T	DnaJ (hsp40) gene. It functions together with DnaK in protein fold. In bacteria, it may influence the formation of biofilm, and is also a novel virulence protein.	Oxidative stress and virulence modulation to the male hosts.	
wCsolGM000671T	DnaK (Hsp70). Modulating polypeptide folding, degradation and translocation across membranes, and protein-protein interactions.		
wCsolGM000670T	hscB. It is the co-chaperone of hsp40, involved in the maturation of iron–sulfur cluster-containing proteins. It seems to help targeting proteins to be folded toward HscA.		
wCsolGM000573T	Toxic component of a toxin–antitoxin (TA) module. It is a very important effector of bacterial-host interaction, especially in stress.		
wCsolGM000052T	Isoprenoid biosynthetic process. It plays important roles in host-pathogen interaction (virulence potential and immune stimulation).		
wCsolGM000968T	Cell cycle-aspartyl protease family protein; polypeptide hydrolase activity.	Cell cycle, blocking of cell division in male hosts.	
wCsolGM000648T	Coproporphyrinogen oxidase, function in heme biosynthesis.		
wCsolGM000846T	CtrA. It controls cell cycle programs of chromosome replication and genetic transcription, with downregulation causing the block of cell division.		Down-regulated
wCsolGM000813T	Actin binding; cytoskeletal protein binding.	Increased transfer in female hosts’ cytoskeleton.	
wCsolGM000584T	Ribosome biogenesis		
wCsolGM000925T	GreA gene. It is not only a transcription elongation factor that promotes transcription fidelity, but also a chaperone to enhance the resistance to heat shock and oxidative stress.	Increased transcription fidelity or enhanced resistance to oxidative stress in female hosts.	

## Data Availability

The RNA sequence data presented in this study are openly available in NCBI with the accession IDs of SRR7031447, SRR7031448, SRR7031449, SRR7031450, SRR7031445, SRR7031446, SRR7031443, SRR7031444, SRR7031441, SRR7031442, SRR7031439, and SRR7031440.

## Supplementary Materials

The following are available online at https://www.mdpi.com/2076-2607/9/2/288/s1, Table S1: Primers used in the qPCR validation of differentially expressed *Wolbachia* genes, Table S2: Biological processes of GO enrichment analysis on all the nine groups of *Wolbachia*-infection associated DEGs, Table S3: Biological processes of GO enrichment analysis on all the nine groups of gender associated DEGs.
