# Supplementary material for: Transcriptomic Analysis Reveals the Sexually Divergent Host–Wolbachia Interaction Patterns in a Fig Wasp
Source: Microorganisms. 2021 Jan 31;9(2):288. doi: 10.3390/microorganisms9020288 (PMC7912686; doi:10.3390/microorganisms9020288)
Supplement: Supplementary file 1 [file microorganisms-09-00288-s001.zip › supplementary files/Table S1.docx]

Table S1. Primers used in the qPCR validation of differentially expressed *Wolbachia* genes

| Gene IDs | Forward primer | Reverse Primer |
| --- | --- | --- |
| wCsolGM001140 | TGGCAAGCCAAACGTAGTCT | ACCGCTTGACTTACAACCGT |
| wCsolGM000515 | GCTAGAAAGCATAGTGGATCGT | GCGACTTCGATTTTCCCTTCA |
| wCsolGM000536 | TTTATCTTCTGCGCCCCGAT | CTCCGTTTTGCAACATCGCT |
| wCsolGM000349 | ACAGAAAAGCCCGAGCGTAA | ACAGAAATCTCTCTTGCGGTGT |
| wCsolGM000223 | TTTGGTGGAGGATTCGGTGG | TCTAATCCTACCGCTTCCCTG |
| wCsolGM000670 | AAAGTCACCGCTAAAACGTGC | ATCATCGCAGTCCAGCAAAT |
| wCsolGM000573 | TTGTTCCTTGGTGCAAAGCC | ACAGCTTTTATCCAACCTTCGT |
| wCsolGM000968 | CTTGATACTGGAGCAACCGA | GGTATTTGGACCACTCCAGC |
| wCsolGM000846 | TGCCTGGTGATATTGACGGA | AGGATGGCCTTTAGTACGACG |
| wCsolGM000813 | CCTCAACAACCTCCGCTAACA | ATCACGCTCTTCCTTGCTCAT |
| wCsolGM000925 | ATGACGAGGGAAGGTTTTG | ACATATTCCACTTCACCGTT |
| wCsolGM000197 | CTATGGGCCGGTTGTGACTT | CCAAGCGCAATTGGGAGATT |
| wCsolGM000202 | AGCCATGGTCTTGGGTAATATAGG | CCACTGTAGCTAGTACCATAACCT |
| wCsolGM000458 | GCAACTTCTGGTCCATTGCT | TCCAAGCCGTGTAGTTGCTC |
| wCsolGM000829 | CTTGACCCAGGGAACAATGA | TCAAACGCTATTGAGCCAAAGA |
| wCsolGM000648 | TGCAGCGCATATGAACACAA | AAGGCTTCACCTACTGCTTT |
| wCsolGM000507 | TTGAACCTAACTGTCCGCCC | AGTTTCAGGAGTGCAGCGAA |
| wCsolGM000579 | GAAACAAGCGCAAGAGATGC | GGTAAACCCATCATACCTGCGA |
| wCsolGM000584 | GTCCGAATGTTGGAAAATCA | GCTCAATACCAACTAGGGAG |
| wCsolGM001038 | CTGCACTCATTGTCCATGCC | AAGGCATCGCGTACTTTACCA |
| wCsolGM000671 | AGAGAAAAGACGGCCAAGCA | AGAACTGATTGCCTGCTCGAA |
| wCsolGM000610 | TGGTGTGACGATGGTGACTT | CGAGCCATCACATTTTGCCT |
| wCsolGM000433 | TTTCGTGTCGGAGCCGGTTA | GCAACATCACCATCAGAGTGC |
| wCsolGM000918 | TTGCGAAGTTGATAAGCGCA | CCCAGCAATAGGCAACCGA |
| wCsolGM000920 | TACGCTGGGGCAAAATGTGG | ACCCTGTACGAAACCCTGTT |
| wCsolGM000052 | GGGAAGCTGTGGGAGATTGA | ACTGGCTTGCTATCTACGCT |
| *groEL* | CAACCTTTACTTCCTATTCTTG | CTAAAGTGCTTAATGCTTCACCTTC |
